# Applying Deep Learning to Continuous Bridge Deflection Detected by Fiber Optic Gyroscope for Damage Detection

**DOI:** 10.3390/s20030911

**Published:** 2020-02-08

**Authors:** Sheng Li, Xiang Zuo, Zhengying Li, Honghai Wang

**Affiliations:** 1 National Engineering Laboratory for Fiber Optic Sensing Technology, Wuhan University of Technology, Wuhan 430070, China; wanghh@whut.edu.cn; 2 School of Information Engineering, Wuhan University of Technology, Wuhan 430070, China; zuoxiang@whut.edu.cn (X.Z.); zhyli@whut.edu.cn (Z.L.)

**Keywords:** bridge damage detection, fiber optic gyroscope, deep learning, convolutional neural network

## Abstract

Improving the accuracy and efficiency of bridge structure damage detection is one of the main challenges in engineering practice. This paper aims to address this issue by monitoring the continuous bridge deflection based on the fiber optic gyroscope and applying the deep-learning algorithm to perform structural damage detection. With a scale-down bridge model, three types of damage scenarios and an intact benchmark were simulated. A supervised learning model based on the deep convolutional neural networks was proposed. After the training process under ten-fold cross-validation, the model accuracy can reach 96.9% and significantly outperform that of other four traditional machine learning methods (random forest, support vector machine, k-nearest neighbor, and decision tree) used for comparison. Further, the proposed model illustrated its decent ability in distinguishing damage from structurally symmetrical locations.

## 1. Introduction

Dynamic modal analysis has been the most commonly used approach for structural damage detection in civil engineering [1,2,3]. The use of wavelet, Hilbert–Huang transform, and other signal processing methods are also the conventional choices for structural damage detection that directly analyze the perturbation of vibration signals [4]. Various structural non-destructive testing approaches [5,6,7] are also significant means for detecting structural damage. Over the last decade, machine-learning algorithms have been used to address a wide range of vibration-based damage detection problems [8,9]. Although most of these techniques are based on vibration responses and such approaches still dominate the diagnosis and prognosis of structural health monitoring [10], feature extraction processes heavily relying on handcrafted intervention prior to damage classification [11] have often become major challenges that limit the effectiveness of various methods.

With the ability of automatic feature extraction and classification, deep convolutional neural networks (CNN) have been explored to address the range of difficulties in such following areas as computer vision [12,13], speech recognition [14], natural language processing [15], medical image processing [16], pathological signal classification [17,18], mechanical fault diagnosis [19,20,21], impact evaluation of natural disasters on infrastructure systems [22], and structural damage detection [23,24,25].

Most of the research efforts on deep CNN-based structural damage detection are essentially associated with the supervised learning processes. In this emerging area, Cha et al. [23] pioneered the deep CNN study of damage detection for cracks in concrete structures, and subsequently, Cha et al. [26] further expanded the detection objectives of structural damage based on the faster Region-CNN (R-CNN). The recent study based on R-CNN to quantify the identified concrete spalling damage in terms of volume was reported in [27]. Xue and Li [28] established a fully convolutional neural networks model to classify the concrete tunnel lining defects. Other image-based researches on structural damage detection using deep learning were reported in [29,30,31]. In addition to two-dimensional convolution operations on structural images, one-dimensional convolution operations which usually spend a considerably cheaper computational cost than that of recurrent neural networks [32], are employed by researchers to perform the signal-based structural damage detection. The structural vibration signal as a typical type of one-dimensional time series [33] data is used to perform deep CNN-based structural damage detection. For instance, Abdeljaber et al. [18] proposed a method for detecting structural damage using one-dimensional CNN for multi-nodal vibration testing of steel frames. Lin et al. [25] simulated the vibration response of simply supported beams under various damage scenarios and proposed a procedure for detecting the categories of structural damage using the one-dimensional CNN. Huang et al. [34] analyzed the mechanical operation process through vibration signal by constructing a one-dimensional CNN.

Although deep CNN technology to some extent relieves the heavy pre-processing on the raw data or feature crafting for the damage detection when using vibration signals, the analysis of bridge damage detection is more complex comparing to that of simple structures, which needs more support of structural responses. In other words, compared with the amount of the degree of structural freedom, the scale of available vibration sensors used for bridge structural damage detection are often finite or even insufficient. To obtain as much structural dynamic information as possible in the case of limited measurement points, sensor optimization layout [35] is generally considered, which results in a decrease in damage detectability of complex structures. Therefore, a novel type of structural response which can easily cover the whole and local test requirement and provide enough structural information for the analysis of damage detection by using deep CNN should be attempted and explored. In this paper, we aimed at the multi-dimensional type of signal and chose a test technique for continuous curve mode of deformation based on fiber optic gyroscope (FOG) to produce continuous deflection of bridge. Detailed fundamental principles of the FOG-based testing technique were reported in [36,37,38]. A corresponding sample set based on supervised learning techniques was established, and a specific one-dimensional CNN model was proposed to automate feature extraction and classification. Specifically, the scheme procedure for the production of structural damage scenarios based on deformation responses was elaborated. The deformation responses and corresponding output labels were established through data augmentation and pre-processing. Furthermore, architectures and algorithms of the proposed one-dimensional CNN, and the partition rules of the dataset used for method verification were discussed. Finally, the performance of the proposed approach was compared with the results of other pattern recognition methods, all of which were conducted under the ten-fold cross-validation [39].

## 2. Design and Implementation of Structural Damage Scenarios

### 2.1. Experimental Platform and Instrumentation

**Figure 1:**
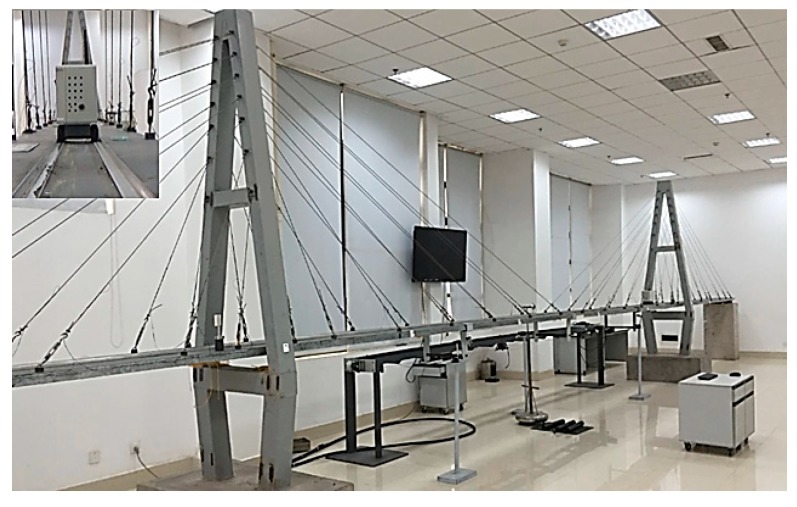
Experimental platform for testing continuous deflection.

**Figure 2:**
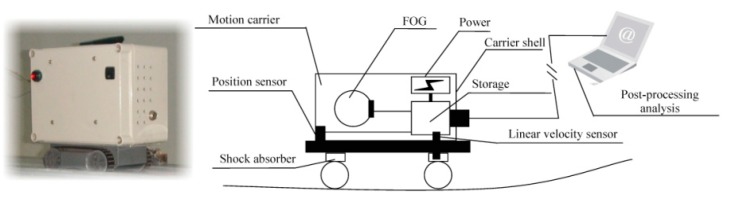
Measuring device integrated in a motion carrier.

A scale-down model of cable-stayed bridge was used as the experimental platform to represent the responses due to the simulation damage. The model shown in Figure 1 with the main span of 9.7 m, tower height of 3.46 m, deck width of 0.55 m, and 56 stay cables, was manufactured at a scale of 1:40. Figure 2 further illustrated a structure diagram and a physical structure of the device dedicated to measuring continuous deflection of the bridge model, which was triggered by a remote controller to perform mobile measurement. When the test device [36] moved along the surface of the bridge model, data of continuous deflection can be collected at sampling rate 150 Hz. Since the measurement period based on the motion carrier was relatively short, the continuous deflection obtained chronologically at each time was regarded as a multi-dimensional variable acquired at the same moment, and the deflection of main span was chosen as the structural deformation response used for subsequent analysis.

### 2.2. Experimental Design and Procedures

The change in structural geometry can reflect a certain degree of transformation of interior mechanical properties. Further, structural damage is one reason for the change of interior mechanical properties of structure. Therefore, the different damage scenarios of the structure theoretically have corresponding structural deformation states. Continuous deflection can provide the dense deformation information, which can present more abundant structural response information than other finite point-based geometry measurement methods [40,41,42]. In the context of an experiment based on supervised learning, it was assumed that the change in the continuous deflection of bridge was only due to the result of structural damage. A metal pad (42.8L × 12.8W × 0.2H cm) with slope at both ends was used to simulate structural deformation caused by damage rather than physically destroying the structure [43]. The pad as an obstacle was placed on the movement path of the measuring device to simulate the deformation caused by structural damage. Compared with the situation without the pad, the measuring device can capture responses of the continuous deflection of bridge under the disturbance of the pad. This localized continuous deflection caused by the influence of the pad was clearly the most important of the continuous deflection of the entire bridge. Using such local responses instead of the global deflections can undoubtedly simplify the training process of the following supervised learning algorithm.

**Figure 3:**
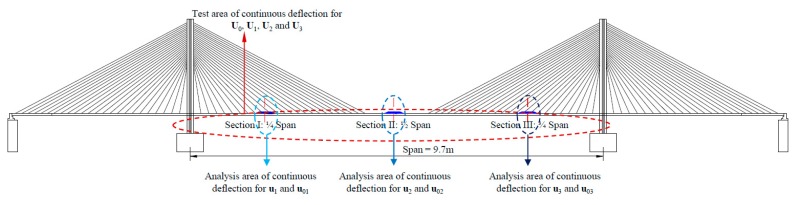
Locations of the metal pad used to assist in simulated damage scenarios.

By this way, as shown in Figure 3, when the pad was not placed, the corresponding continuous deflection of the bridge was defined as $U_{0}$. For each of the three damage scenarios, one pad was placed at a position each time, and therefore, $U_{1}$, $U_{2}$, and $U_{3}$ can be obtained. Here, $U_{0}$, $U_{1}$, $U_{2}$, and $U_{3}$ as raw data of continuous deflection represented four types of simulated structure states, respectively. To improve the training efficiency and save the computational overhead of the supervised learning, $U_{1}$, $U_{2}$, and $U_{3}$ were truncated to $u_{1}$, $u_{2}$, and $u_{3}$. Such truncated selection in the areas affected by the pad can be estimated through both the original testing curves and the dimension of pad in the context of experiment based on supervised learning. In the intact and three damage scenarios, the actual benchmarks of $u_{1}$, $u_{2}$, and $u_{3}$ were $u_{01}$, $u_{02}$, and $u_{03}$, respectively. A common baseline for the three damage scenarios was defined to facilitate analysis. The weights of $u_{01}$, $u_{02}$, and $u_{03}$ were regarded as equal and their average $u_{0}$ was designated as the nominal benchmark of $u_{1}$, $u_{2}$ and $u_{3}$. The following work utilized $u_{0}$, $u_{1}$, $u_{2}$, and $u_{3}$ to conduct the damage detection based on deep CNN algorithm.

### 2.3. Raw Samples of Continuous Deflection of Bridge

**Figure 4:**
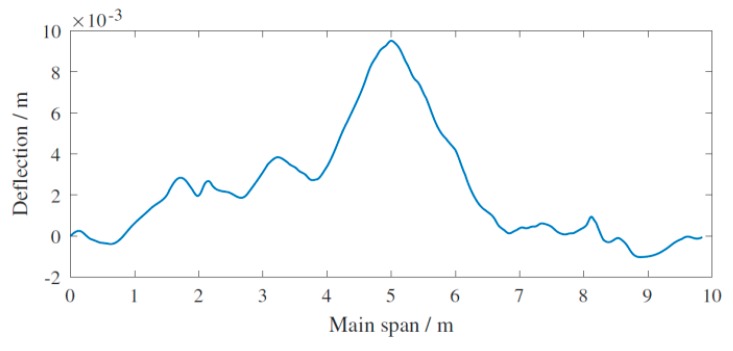
Deformation of main span depicted by continuous deflection.

The spatial resolution in motion direction refers to the adjacent sampling interval of the device in Figure 2. This parameter is determined by the wheel diameter and the reticle of the rotational speed code wheel, and is approximately 1.48 mm. For the continuous deflection of the main span, taking intact scenario for an example shown in Figure 4, the deformation response of $U_{0}$ consisted of sequence data of 6554 dimensions which depicted the length of main span of 9.7 m. Due to the high spatial resolution, the continuous curve clearly reflected a certain degree of pre-camber applied at the main span. Moreover, the continuous curve revealed that the experiment platform did not exhibit completely symmetrical structural deformation owing to the handcrafted control for the cable force.

**Figure 5:**
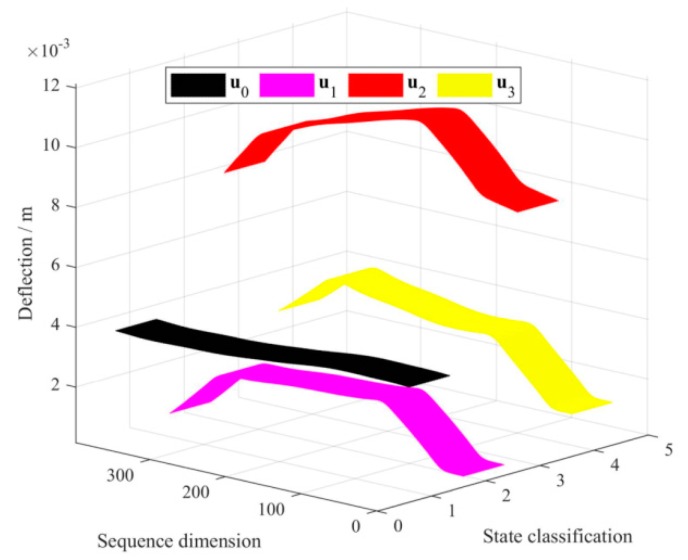
Truncated deformations depicted by continuous deflections.

The local continuous deflection curves of $u_{0}$, $u_{1}$, $u_{2}$ and $u_{3}$ were shown in Figure 5. Each type of the local continuous deflection curve contained 390 dimensions of sequence data. The coverage length of the area affected by the pad was considered to be the primary basis for determining the length of the local continuous deflection. Moreover, through preliminary data observation, the length of the region having the largest influence range among the three disturbance positions of the pad was selected, rounded, and defined as the final truncated length, which guaranteed the consistency of multiple sets of sample dimensions. The continuous curve mode test technique was used to separately collect the structural response of the scale-down bridge model under intact and simulated structural damage, and five groups of $U_{0}$, $U_{1}$, $U_{2}$, and $U_{3}$, were collected, respectively. Therefore, five groups of $u_{0}$, $u_{1}$, $u_{2}$, and $u_{3}$ corresponded to four types of structural conditions, namely intact, damage_1/4_, damage_1/2_, and damage_3/4_, and these were used as raw samples to conduct the following study.

## 3. Detection Methodology Based on Deep CNN

### 3.1. Data Augmentation and Pre-processing

**Figure 6:**
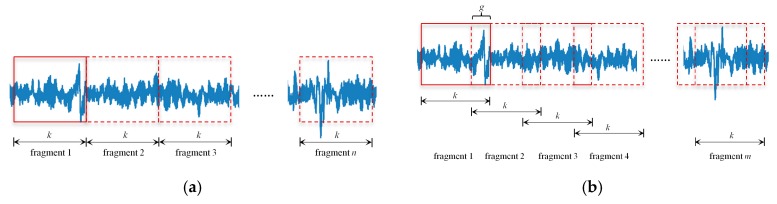
Data augmentation of (**a**) common means and (**b**) adopted operation.

Data augmentation and pre-processing are two essential tasks before carrying out deep learning. The former is always the first choice to boost the performance of a deep network. For image recognition based on deep CNN, there are a wide range of ways to perform data augmentation [12,44,45]. However, the above approaches are not suitable for signal-based pattern classification when using deep CNN algorithm. As shown in Figure 6a, dividing the raw acquisition signals into the same sub-fragment directly is common means of data augmentation [24,25]. It can be seen from Figure 6b that, for fragments of the same length as that in Figure 6a, the overlapping zone set in the adjacent fragments causes the amount of the fragment m to be larger than n shown in Figure 6a, which effectively increases the amount of data size.

**Figure 7:**
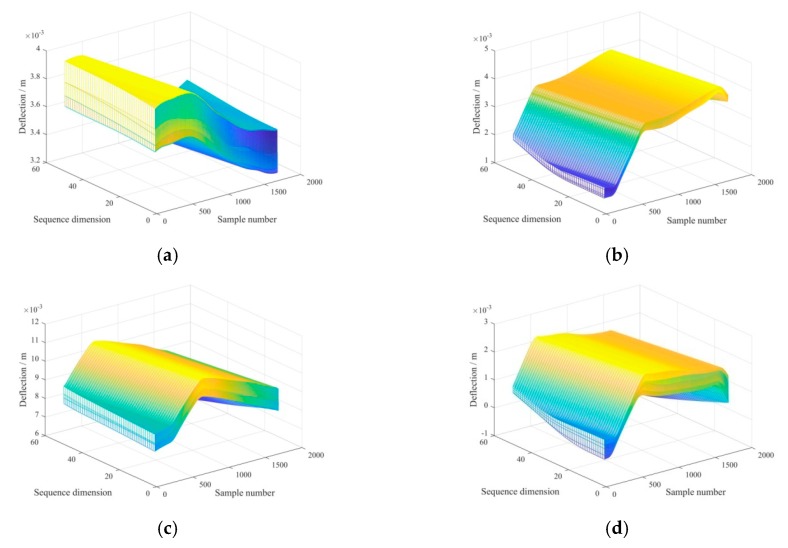
Sample set represented by mesh graphics through data augmentation for (**a**) intact, (**b**) damage_1/4_, (**c**) damage_1/2_, and (**d**) damage_3/4__._

Since the original experimental samples were small, the overlapping zone was taken as g = 1. It was obvious that the larger the value of k in Figure 6, the smaller the number of fragments after data augmentation and vice versa, which also indicated the greater number of fragments needed more computational overhead of model training. With the consideration of a tradeoff result between the training objective of model and the computational overhead, the length of fragment was set as k = 50, followed by the 390-dimensional original sequence becoming 341 50-dimensional sequence samples. For the five raw groups of $u_{0}$, $u_{1}$, $u_{2}$, and $u_{3}$, after data augmentation, the sample set of $u_{0}^{\prime }$, $u_{1}^{\prime }$, $u_{2}^{\prime }$, and $u_{3}^{\prime }$, each including 1705 samples, corresponding to Figure 7a–d, were shown by mesh graphics.

To eliminate the difference in the deflection amplitudes of four types of samples in Figure 7 and boost a better classification effect [46,47], a type of min-max normalization [48] expressed in Equation (1) is used to normalize all the amplitudes to the range of 0~1.

(1) $$u_{i}^{\prime \prime }=\frac{u_{i}^{\prime }-min(u_{i}^{\prime })}{max(u_{i}^{\prime })-min(u_{i}^{\prime })},\left(i=0,1,2,3\ldots \right)$$

As shown in Table 1, raw and truncated represented the continuous deflections of the test area and analysis area shown in Figure 3, respectively. After data augmentation and normalization based on the truncated stage, each category of the four-dataset including intact and three types of simulated damage contained 1705 samples. The four types of state, namely, $u_{0}^{\prime \prime }$, $u_{1}^{\prime \prime }$, $u_{2}^{\prime \prime }$, and $u_{3}^{\prime \prime }$ were used as input data, in which $u_{0}^{\prime \prime }$ represented the intact baseline and the rest three represented different damage scenarios. One-hot form was used to describe the output labels corresponding to the four categories, meaning that the label vector was generated by the rule that the vector had all zero elements except the position j, where j was the type number of structural state.

The entire measurement process was performed under stable temperature field, and the data of this study came from actual measurements, which already contained noise disturbances existing in the indoor environment. Therefore, extra interferences of simulated noise and temperature effect were not further considered here.

### 3.2. Descriptions of the Proposed CNN Architecture

**Figure 8:**
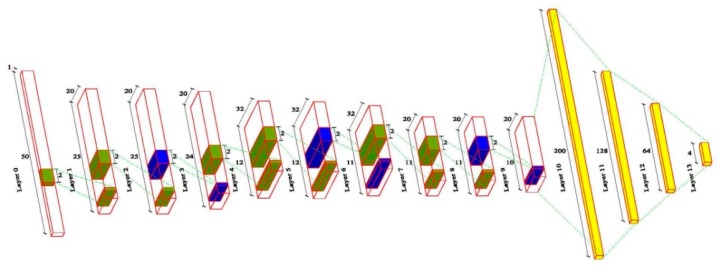
The proposed deep CNN architecture.

Table 2 gave the details of the proposed CNN structure through the trial-and-error under the current computing resource configuration. The model structure was inspired by Cifar-10 [49], in which operations of convolution and pooling were not pairwise used. Figure 8 showed the graphical representation of CNN structure with 50 input sample lengths where the green, blue, and yellow referred to the kernel size, max-pooling, and fully connected layer, respectively.

Layer 0 as the input layer in Figure 8 was convolved with a kernel of size 2 to produce Layer 1. The convolution and cross-correlation were used interchangeably in deep learning [50], which can be described as:

(2) $$f(i)=\sum_{n=1}^{N}s(i+n)k(n)$$

where s is input signal, k is filter, and N is the number of elements in s. The output vector f is the cross-correlation of s and k. Next, Layer 1 was convolved with a same kernel size to produce Layer 2. After two times of convolution, a max-pooling of size 2 was applied to every feature map (Layer 3). By repeating the above operations two times, other four convolutional layers and two max-pooling layers were created. In Layer 9, the neurons were then fully connected to 200 neurons in Layer 10 by flatten. Eventually, Layer 10 was fully connected to 128 neurons in Layer 11 and Layer 11 was fully connected to 64 neurons in Layer 12. Finally, Layer 12 was connected to the last layer (Layer 13) with 4 output neurons which represented intact, damage_1/4_, damage_1/2_ and damage_3/4_.

Because the gradient of the left side of rectified linear unit (ReLU) [51] as shown in (3) is always zero, the activation operation may become invalid during training process if the weights updated by a large gradient become zero after being activated.

(3) $$f(x)=\{\begin{array}{c} \begin{array}{cc} x, & x\geq 0 \end{array}\\ \begin{array}{cc} 0, & x<0 \end{array} \end{array}$$

The Leaky ReLU method [52] is a good alternative to address such problem by considering a parameter α in (4),

(4) $$f(x)=\{\begin{array}{c} \begin{array}{cc} x, & x\geq 0 \end{array}\\ \begin{array}{cc} \alpha x, & x<0 \end{array} \end{array}$$

where α is usually set to a small number, and once α is set, its value will keep constant. This allows a small, non-zero gradient when the unit is not active. The parametric rectified linear unit (PReLU) [53] which has the same mathematical expression to Leaky ReLU, takes this idea further by making the coefficient α into a parameter that is learnt along with the other neural network parameters. Since it was not necessary to consider how to specify α, PReLU was used to take the place of Leaky ReLU in this work as an activation function for the convolutional layers (1, 2, 4, 5, 7 and 8) and two fully connected layers (11 and 12).

Further, the Softmax function was used to compute the probability distribution of the four output classes, which can be expressed as follows:

(5) $$p_{k}=\frac{e^{x_{k}}}{\sum_{i=1}^{n}e^{x_{i}}}$$

where $x_{k}$ is the input of last layer, n is the number of output nodes and output values of $p_{k}$ are between 0 and 1 and their sum equals to one. Equation (5) was used for Layer 13 in Figure 8 to predict which category the input signals (intact, damage_1/4_, damage_1/2_, or damage_3/4_) belonged to.

Compared with shallow neural networks, deep CNN as a more complicated model contains more hidden layers and more weights, and is particularly prone to overfitting. In the proposed deep CNN, a dropout rate of 0.35 was used before the classification layer (Layer13) as shown in Table 2, which together with early stopping [54] mentioned in the following, effectively suppressed incidence of overfitting during all training processes.

### 3.3. Training Setting

Ten percent of the total dataset was used for test, while the rest 90% was divided into two parts, namely, training (80%) and validation (20%). The reason for validation was to evaluate the performance of the model for each epoch and prevent overfitting.

Because the cross entropy function is much more sensitive to the error, the learning rules derived from the cross entropy function generally yield better performance. Here, categorical cross entropy was used as the objective function to estimate the difference between original and predicted damage types, expressed as follows:

(6) $$J=\sum_{i=1}^{k}\left[-d_{i}ln(y_{i})-(1-d_{i})ln(1-y_{i})\right]$$

where J is the cross entropy, $y_{i}$ is the output of prediction class, $d_{i}$ is the original class in the training data, and k is the number of output nodes.

To minimize the above objective function, adaptive moment estimation (Adam) was selected as the optimization algorithm. It calculated an adaptive learning rate for each parameter and stored both an exponentially decaying average of past squared gradients and an exponentially decaying average of past gradients [55]. Details about the training parameters in this work are given in Table 3, in which the early stopping technique was used to control training epochs and further avoid overfitting, and the parameters set in Adam were based on the suggestion in [56].

Moreover, a ten-fold cross-validation approach was used in this study, the purpose of which was to reduce the sensitivity of algorithm performance to data partitioning and to obtain as much valid information as possible from the enhanced data. First, all the prepared dataset was randomly divided into ten equal parts. Nine out of ten parts of the total were used to train the proposed deep CNN while the remaining one-tenth dataset were used to test the performance of the model. This strategy was repeated ten times by shifting the training and test dataset. The accuracies reported in the paper were the average values obtained from ten evaluations.

## 4. Results and Discussion

**Figure 9:**
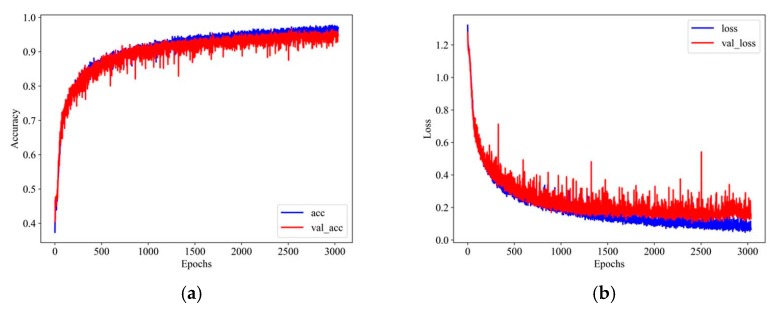
Typical training processes about (**a**) accuracy and (**b**) loss.

The proposed CNN model was implemented by Python package Tensorflow and Keras [57]. The average training runtime of each fold for the proposed model was approximately 15 minutes, which was run on a GPU core (GTX 1080 Ti) with twelve 2.20 GHz processors (Intel Xeon E5-2650 v4). According to the setting in Table 3, the training processes showed that in the initial 500 epochs, the convergence speed was rather quickly for all of the dataset from the ten-fold cross-validation, but it still needed approximately 3000 to 4500 epochs to reach the best performance based on the patience rule set in early stopping. The typical training process regarding accuracy and loss represented by fold 2 is shown in Figure 9, which stopped at the epochs of 3036.

**Figure 10:**
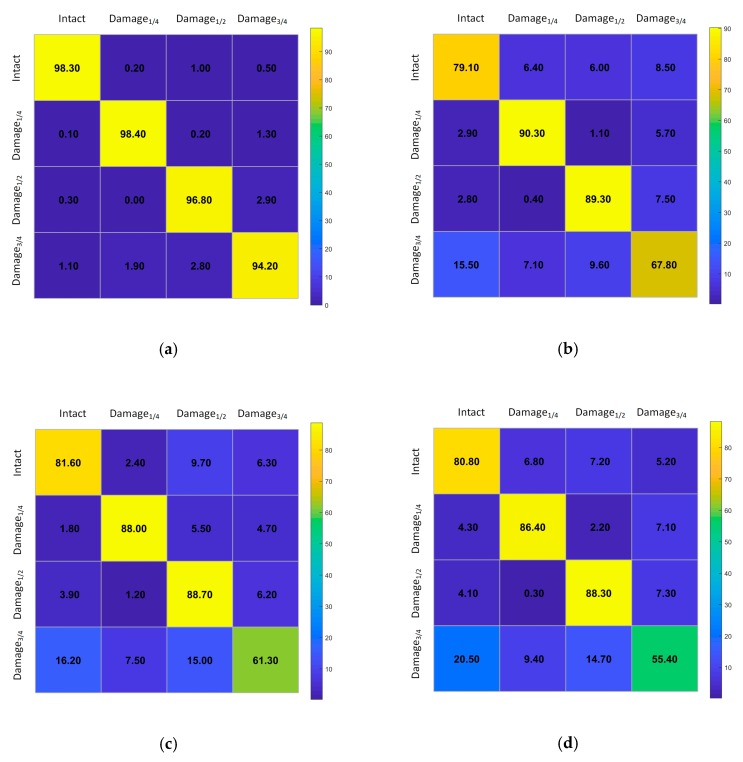

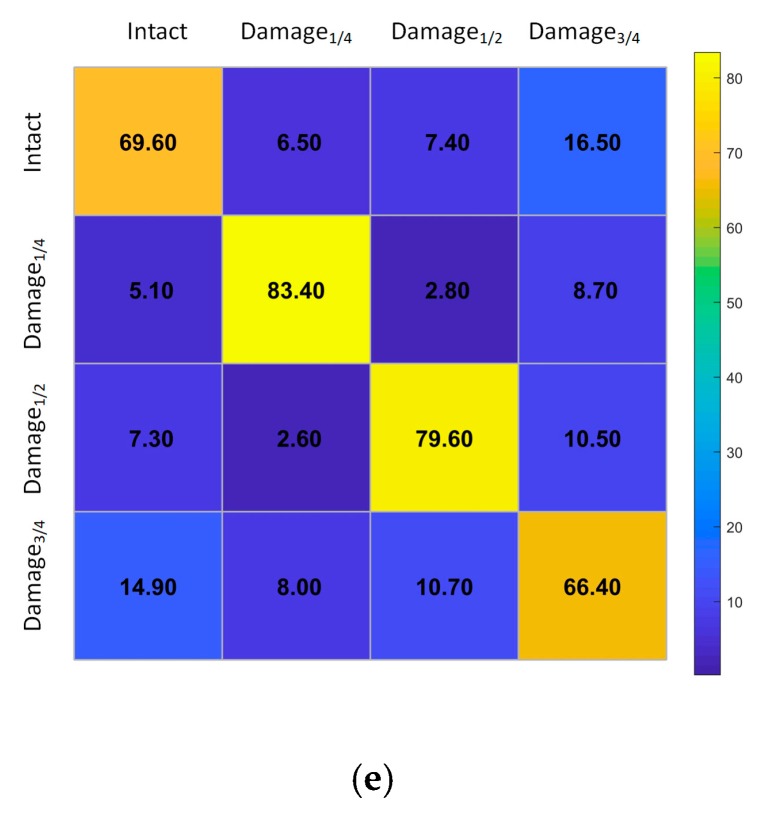
The confusion matrices of (**a**) CNN, (**b**) random forest, (**c**) support vector machine, (**d**) k-nearest neighbor, and (**e**) decision trees.

The confusion matrix cross all ten-fold was presented in Figure 10a. It was observed that 98.3% of $u_{0}^{\prime \prime }$ signals were correctly classified as intact. Moreover, 1.7% of $u_{0}^{\prime \prime }$ were erroneously classified as other damage categories. Further, a high percentage of 98.4% of $u_{1}^{\prime \prime }$ signals were correctly classified as damage_1/4_ with 1.3% of $u_{1}^{\prime \prime }$ wrongly classified as damage_3/4._ For $u_{2}^{\prime \prime }$ the accuracy rate for damage_1/2_ reached 96.8% with 2.9% of $u_{2}^{\prime \prime }$ wrongly predicted as damage_3/4_. Similarly, 94.2% of $u_{3}^{\prime \prime }$ signals were correctly classified as damage_3/4_ with 5.8% wrongly classified as intact (1.1%), damage_1/4_ (1.9%), and damage_1/2_ (2.8%).

**Figure 11:**
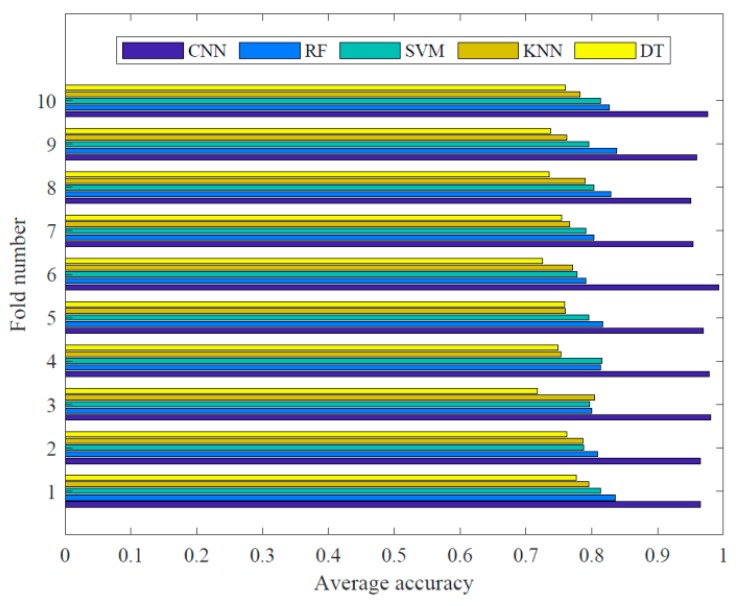
Comparisons of average accuracy in each fold of cross-validation.

Furthermore, to evaluate the capability in each fold of cross-validation, average accuracy results shown in Figure 11 for different classes were compared between the proposed model and other four pattern recognition methods. When the samples were directly used to classify without heavy consideration regarding features extraction, the accuracy of automatic detection for proposed CNN model (96.9%) was obviously better than that of random forest (RF) (81.6%), support vector machine (SVM) (79.9%), k-nearest neighbor (KNN) (77.7%), and decision trees (DT) (74.8%). Here, the allocation of dataset of the four comparison methods was consistent with the proposed deep CNN algorithm. To fully compete with the proposed model, the most decent key hyperparameters set in sklearn [58] for RF, SVM, KNN and DT were derived through trial-and-error. To further quantify the effect of classifiers, Figure 10b–e show confusion matrices of the other four methods, respectively. It was observed that the best accuracy in various comparison methods can reach to 90.3% as shown in Figure 10b, which was still inferior to the lowest accuracy 94.2% as shown in Figure 10a.

**Figure 12:**
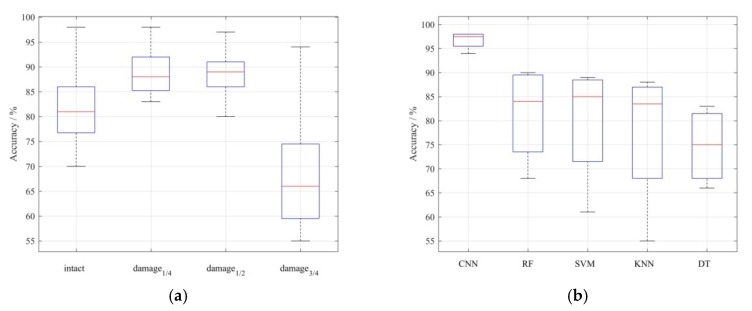
Accuracy distribution based on (**a**) damage category and (**b**) detection method.

Next, as shown in Figure 12a, for all five methods, the classification effects on damage_1/4_ obviously outperformed the results of the other three categories. Moreover, the detection results of damage_3/4_ were the worst in all methods, having a direct influence on the average accuracy of various ways. Further, as shown in Figure 12b, the classification imbalance presented in the confusion matrix was most severe when KNN was used as the classifier. This phenomenon may be related to the relatively lower algorithm complexity of KNN [59,60] compared with other methods mentioned in this work. Only the proposed approach based on deep CNN effectively mitigated this imbalance, although the accuracy of damage_3/4_ in Figure 10a was still slightly less than the other three classes. The limited data samples should be a major aspect for such imbalance. In addition, current tests were all from the one-way results and lack of the data from the opposite direction. This may introduce a cumulative system error to the results of the structural response. Further, only slight pre-processing was carried out for the original dataset, which reduced the learning ability of each method mentioned in this paper. Actually, as shown in Table 4, the other four machine learning methods for comparison had fewer key parameters to consider in the process of balancing training accuracy and training error than the proposed method. This weak complexity, determined by the principles of the algorithm, resulted in a poor predictive effect on training and validation. Therefore, under the same circumstance, the proposed approach clearly demonstrated better overall performance in automatic feature extraction than other comparison means.

## 5. Conclusions

A deep learning CNN model with 11 trainable hidden layers was proposed to automatically extract and classify the bridge damage represented by the continuous deflection of bridge. Although current research on the use of FOG-based test technique to detect the damage of a scale-down bridge model through deep learning is just a pilot study, the following conclusions can be drawn:

(1) In the case where it is easy to measure the FOG-based continuous deflection of the target structure, it is convenient to build structural deformation database that can provide sufficient training samples for deep learning-based damage detection.
(2) Based on the data preparation strategies adopted in this work, one-dimensional convolution operation can effectively extract the detailed features of bridge deflection after a slight data pre-processing.
(3) The deep CNN-based method as a classifier has at least 15.3% accuracy advantage over other traditional methods mentioned in this paper in distinguishing different types of bridge deformation modes.
(4) Even if the same level structural damage occurs at a symmetrical position, the proposed method can still achieve satisfactory results with a deviation of only 4.2% for the recognition accuracy of damage at the symmetrical position.
(5) For an actual bridge with a complete deformation monitoring database, the advantage of deep learning on automatic extracting of features of large-scale database can be exploited to search the damage or provide the preliminary diagnostic findings. Moreover, since the FOG-based measurement system has higher test accuracy for larger distributed deflections [61], the proposed method should be more suitable for long-span bridges.

## Figures and Tables

**Table 1:** The dataset details of training and test.

Processing Stage	Variable				Dimension of Each Variable	Total Samples
Raw	$U_{0}$	$U_{1}$	$U_{2}$	$U_{3}$	6554	4 × 5 = 20
Truncated	$u_{0}$	$u_{1}$	$u_{2}$	$u_{3}$	390	
Augmentation	$u_{0}^{\prime }$	$u_{1}^{\prime }$	$u_{2}^{\prime }$	$u_{3}^{\prime }$	50	4 × 1705 = 6820
Normalization	$u_{0}^{\prime \prime }$	$u_{1}^{\prime \prime }$	$u_{2}^{\prime \prime }$	$u_{3}^{\prime \prime }$		

**Table 2:** The details of CNN structure.

Layers	Type	No. of Neurons (Output Layers)	Kernel Size	Stride	Padding	Activation
0-1	Convolution	25 × 20	2	2	Same	PReLU
1-2	Convolution	25 × 20	2	1	Same	PReLU
2-3	Max-pooling	24 × 20	2	1	Valid	——
3-4	Convolution	12 × 32	2	2	Same	PReLU
4-5	Convolution	12 × 32	2	1	Same	PReLU
5-6	Max-pooling	11 × 32	2	1	Valid	——
6-7	Convolution	11 × 20	2	1	Same	PReLU
7-8	Convolution	11 × 20	2	1	Same	PReLU
8-9	Max-pooling	10 × 20	2	1	Valid	——
9-10	Flatten	200	——	——	——	——
10-11	Dense	128	——	——	——	PReLU
11-12	Dense with dropout	64	——	——	——	PReLU
12-13	Dense	4	——	——	——	Softmax

Note: PReLU—parametric rectified linear unit.

**Table 3:** The training parameters of CNN structure in this work.

Batch Size	Epoch	Patience in Earlystopping	Adam			
			Initial Learning Rate	$\beta_{1}$	$\beta_{2}$	ε
128	5000	500	0.001	0.9	0.009	1.0 × 10^−8^

**Table 4:** Key parameters set in the comparison methods.

RF	SVM	KNN	DT
FS criteria = gini Number of DT = 150	Kernel = rbf gamma = 10 *C* = 10	*K* = 6 DM = euclidean	FS criteria = entropy

Note: FS—Feature selection, gamma—The influence of kernel radius, C—The penalty parameter, *K*—k value defined in KNN, DM—Distance metric.
